# Zr-based metal–organic frameworks for colorimetric sensing applications

**DOI:** 10.1039/d5lf00378d

**Published:** 2026-03-12

**Authors:** Xinhao Li, Sergey M. Borisov, Francesco Carraro, Paolo Falcaro

**Affiliations:** a Institute of Physical and Theoretical Chemistry, Graz University of Technology Stremayrgasse 9 Graz 8010 Austria paolo.falcaro@tugraz.at; b Institute of Analytical Chemistry and Food Chemistry, Graz University of Technology Stremayrgasse 9 Graz 8010 Austria

## Abstract

Zirconium-based metal–organic frameworks (MOFs) possess high porosity, chemical stability, and chemical/structural tunability, making them versatile platforms for colorimetric sensing. This focused review highlights Zr-based MOF applications for the detection of acids and bases, aqueous ions, biomolecules, and volatile and gaseous analytes, discussing the mechanisms of colorimetric response, including host–guest interactions, post-synthetic functional modification, and analyte-induced structural changes. Insights gained from these studies provide guidance for the rational design and fabrication of next-generation MOF colorimetric sensors with enhanced sensitivity, selectivity, and stability.

## Abstract 2

This review highlights Zr-based MOF colorimetric sensing mechanisms and their applications in detecting acids and bases, aqueous ions, biomolecules, and volatile/gaseous analytes.

## Graphical abstract

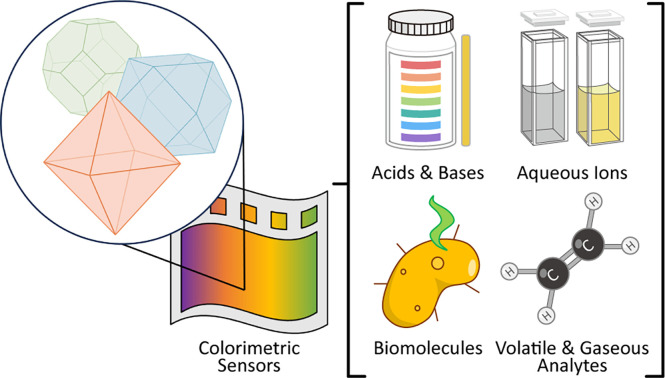

## Introduction

### Colorimetric sensors

A colorimetric sensor can be defined as an optical device that converts physical or chemical information into a detectable colour change.^1,2^ In general, colorimetric sensors, due to their simple operation, affordable cost, portability, and facile signal digitalization, show great potential for applications across multiple fields.^3^ Benefiting from their unique advantages of sensitivity and easy readability, colorimetric sensors enable rapid water quality assessment in environmental monitoring,^4^ real-time detection of pesticide and pollutant residues as well as freshness indication in food safety,^3,5–7^ quick gas detection in industrial production,^8^ and fast monitoring of biomarkers in healthcare.^9^ The global biosensors market was valued at approximately USD 25.5 billion in 2021, and is projected to exceed USD 36 billion in 2026.^10^ Specifically, referring to healthcare, the progress with wearable colorimetric sensors^11^ provides non-invasive monitoring of biological indicators from human sweat.^12,13^ As an important category of biosensors, colorimetric sensors are expected to occupy a significant share of this expanding market, driven by their low cost, ease of use, and applicability in areas such as food safety, environmental monitoring, and point-of-care diagnostics.^14,15^

It should be noted that, according to the Cambridge definition (*i.e.*, chemical sensors are miniaturized analytical devices^16^), many significant works on powders or aqueous dispersions discussed in this focused review article cannot strictly be regarded as sensors. However, we acknowledge that the disclosed sensing properties have the potential to lead to devices *via* subsequent fabrication/integration steps. In practical terms, such developments must ultimately address how sensors are used in real applications. Although numerous systems operate as single-use sensors, users increasingly require devices that can function reliably over extended periods and through multiple cycles. Consequently, advanced sensors should combine reversibility and regenerability, ideally enabled by simple and efficient regeneration procedures.

### Metal–organic frameworks

Metal–organic frameworks (MOFs), as a relatively recent generation of crystalline porous materials, possess high accessible surface area and pore volume, tunable pore environment, and designability.^17^ The well-defined structure of MOFs allows people to customize their functions through rational design or post-synthetic modification^18,19^ or as components in complex composites.^20^ As such, MOFs have become a versatile functional material platform^17^ for numerous applications, including sensing.^21^ Specifically, MOFs hold promise as colorimetric sensors, whether by directly participating in the colorimetric response or by forming hybrid functional materials combined with other functional molecules or materials.^17,22^

Among the different MOF subclasses, for colorimetric sensing, Zr-based MOFs are widely explored. While literature reports widespread assessments on their reproducibility,^23^ different synthetic approaches are currently explored to progress industrial manufacturing.^24–27^ The strong Zr–O bonds endow Zr-based MOFs with a robust framework, making the structure stable to exposure to water, acids, and bases.^23,28–33^ Most Zr-based MOFs feature Zr_6_ clusters as secondary building units (SBUs), which are usually coordinated with organic ligands in 6-, 8- and 12-connected coordination numbers. The high connectivity offers effective protection to the Zr_6_ metal core. The open coordination sites that are not involved in framework formation are usually capped by small molecules such as acetic acid, benzoic acid, or water molecules. Furthermore, these open coordination sites can also be readily modified with functional groups, expanding the application range of some environmentally sensitive functional molecules. In addition, both the ligands and Zr_6_ clusters can serve as adsorption sites for specific analytes, enabling diverse recognition pathways and tunable sensing responses.

The presence of tunable coordination sites and multiple adsorption sites makes Zr-based MOFs highly versatile for sensing applications. These structural features not only enable selective interaction with target analytes but also provide a rational basis for organizing the discussion of their properties and applications in this review. This review is organized into five main sections. The first section examines structural and chemical properties of eight representative Zr-based MOFs, correlating these material properties to their colorimetric sensing performances. The second section focuses on applications in four key fields, such as 1) acids and bases detection, 2) aqueous ion sensing, 3) biomolecule detection, and 4) gas and vapour sensing. Attention is given to the underlying colorimetric mechanisms and fabrication methods. The third section provides an overview of the reported Zr-based MOF colorimetric sensors, including a comparative summary and critical discussion of their target analytes, functional sites, and fabrication strategies. This section also examines the strengths and limitations of different design approaches and identifies the remaining challenges for achieving practical and efficient sensing systems. The fourth section summarizes how MOF particles can be fabricated into functional films, membranes, or patterned structures suitable for practical sensing applications. Finally, a summary and outlook are presented, highlighting future directions for the rational design and practical development of Zr-based MOFs colorimetric sensors.

This review focuses on Zr-based MOFs for colorimetric sensing applications under visible light irradiation, with particular emphasis on systems that generate visible colour changes which can be directly observed by the naked eye. This focus enables a clearer discussion of analyte, functional sites and fabrication methods in relation to practical applications. For readers interested in MOF-based optical sensing strategies that include photoluminescence or fluorescence effects, several recent reviews provide systematic and detailed discussions.^34–38^

## Zr-based MOFs and their structure

**Fig. 1:**
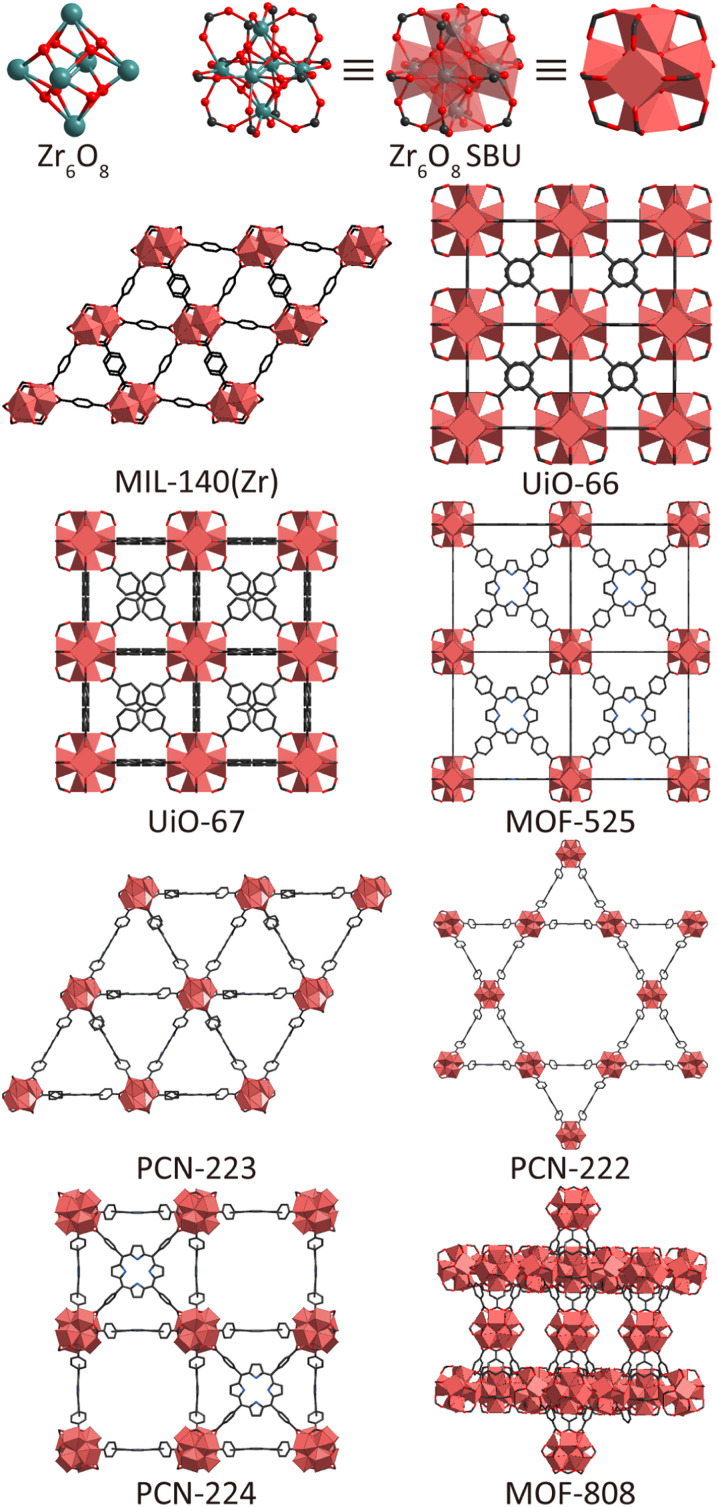
The structures of Zr_6_O_8_ cluster, Zr_6_O_8_ SBU and 8 zirconium-based MOFs. The notation used to describe SBUs is consistent with the one reported by Kaskel.^44^

The Zr-based MOFs applied as colorimetric sensors mentioned in this review belong to eight distinct MOF structures. Among these eight Zr-based MOFs (Fig. 1), MIL-140(Zr) possesses a unique structure. Based on the rod-SBU simplification,^39^ MIL-140(Zr) can be described as exhibiting an underlying **gui** topology, consisting of zirconium oxide chains connected by benzene-1,4-dicarboxylic acid (BDC), forming triangular one-dimensional channels.^40,41^ MIL-140(Zr) is typically synthesized *via* solvothermal methods and the yielded material has a dense framework with a **rod** topology.^42^ As a result, this MOF possesses a lower surface area (450 m^2^ g^−1^), smaller pore size (3.2 Å) and reduced pore volumes (0.20 cm^3^ g^−1^) compared with the other Zr-based MOFs mentioned.^40,41^

In contrast to the unique **rod** topology of MIL-140(Zr), the other seven MOFs are composed of Zr_6_O_8_ metal clusters.^43^ Each Zr_6_O_8_ cluster contains eight oxygen (μ_3_-O) atoms, four of which are generally considered to be hydroxyl groups, coordinating the zirconium atoms at the eight faces of the octahedron.^45,46^ These Zr_6_O_8_ metal clusters connect with the carboxylate groups of organic ligands through three different coordination numbers—6-connected, 8-connected, and 12-connected—forming distinct secondary building units (SBUs) centred on the Zr_6_O_8_ clusters.

Among these Zr_6_O_8_-based MOFs, UiO-66 and its isoreticular framework UiO-67 are representative examples of the 12-connected category and share the same **fcu** topology.^46,47^ Their organic ligands, BDC (UiO-66) and biphenyl-4,4′-dicarboxylic acid (BPDC) (UiO-67), are both linear dicarboxylate ligands with two connectivity points. With the benefit of longer ligands, UiO-67 provides a larger pore than UiO-66, as reflected in their surface areas, pore sizes, and pore volumes (Table 1). Because of the stable 12-connected Zr_6_O_8_ metal cluster, UiO-66 and UiO-67 both exhibit high thermal stability, with decomposition occurring above 400 °C.^47,48^ By employing modulators and deprotonating agents,^47,49^ UiO-66 and its isoreticular frameworks (UiO-67, UiO-68, UiO-66-NH_2_, *etc.*) can be synthesized through modified solvothermal procedures.^50^

**Table 1:** The Zr-based MOFs applied as colorimetric sensors mentioned in this review

MOFs	Topology	Surface area (m^2^ g^−1^)	Pore sizes (Å)	Pore volume (cm^3^ g^−1^)	Ref.
MIL-140(Zr)	**gui**	450	3.2	0.20	40, 41
MOF-808	**pcn**	2060	18.4	0.84	53, 54, 60
PCN-224	**she**	1616	19	1.60	33, 58
MOF-525	**ftw**	1955	18	0.55	30, 52, 59
PCN-222 (MOF-545)	**csq**	1998	17	1.65	31, 59
UiO-66	**fcu**	1187	6, 12	0.46	28, 46, 60
UiO-67	**fcu**	1648	12, 23	0.68	28, 47, 61
PCN-223	**shp**	1600	12	1.10	51

MOF-525 also features a 12-connected metal cluster, constructed from square-shaped *meso*-tetrakis(4-carboxyphenyl)porphyrin (TCPP) ligands, and adopts an **ftw** topology.^30^ Each ligand coordinates with the edges of the Zr_6_O_8_ octahedron metal cluster, thus constructing a stable framework. For example, MOF-525 exhibits good thermal stability (decomposition starting from 400 °C) and acid stability (pH = 5, 24 h), and can be synthesized *via* a solvothermal method at a relatively low temperature (65 °C).^30^

The lattice of PCN-223 is also constructed by connecting TCPP ligands; however, PCN-223 has a **shp** topology.^51^ Its Zr_6_O_8_ metal cluster forms 12 connections through eight di-dentate carboxylates and four mono-dentate carboxylates. The metal clusters are disordered around a threefold rotational axis, exhibiting an unusual Zr_6_ cluster in a 12-connected hexagonal prism mode as the structural node, rather than the octahedral Zr_6_O_8_ cluster found in MOF-525. This topology results in relatively larger pores, with a pore size of 12 Å and pore volume of 1.10 cm^3^ g^−1^. The synthesis of PCN-223 can be achieved *via* solvothermal methods.^50^

By modifying the solvothermal synthesis method, the Zr_6_O_8_ cluster and TCPP ligand can construct PCN-222 (also called MOF-545).^31^ In its framework, the octahedral Zr_6_O_8_ cluster has two carboxylate groups (–COO^−^) linked to each face. This results in the 8-connected category, adopting a **csq** topology and featuring two types of pores with different shapes and sizes—triangular and hexagonal. This pore environment provides a pore size of 17 Å, and a large pore volume of 1.65 cm^3^ g^−1^, enabling the uptake of larger molecules, either as functional sites or analytes. PCN-222 maintains structural integrity and porosity over a wide pH range that includes severe acidic conditions (pH = 2–9).^30^ This level of chemical stability, which is rarely observed in MOFs, is advantageous for practical applications as MOF performance can be retained under a variety of working conditions.

In addition to the 12- and 8-connected categories, the Zr_6_O_8_ cluster and TCPP ligand can also construct PCN-224, which belongs to the 6-connected category. In PCN-224, all carboxyl groups of the TCPP ligand coordinate to the six edges of the Zr_6_O_8_ octahedral cluster that are not perpendicular to the threefold inversion axis, forming PCN-224 with **she** topology.^33^ Due to the lower connectivity, the thermal stability of PCN-224 is slightly lower than that of MOF-525, and decomposition starts from 320 °C. Meanwhile, the lower connectivity affords PCN-224 a larger pore volume (1.60 cm^3^ g^−1^), which is partly occupied by the remaining TCPP ligands in MOF-525.^52^

MOF-808 is also constructed by a 6-connected Zr_6_O_8_ metal cluster. In contrast to PCN-224, all the carboxylate groups from benzene-1,3,5-tricarboxylic acid (btc) coordinate to the six edges that are perpendicular to the threefold inversion axis of the octahedron, forming a **pcn** topology to MOF-808.^53^ Similarly to PCN-224, 6-connectivity offers a considerable pore size (18.4 Å) and pore volume (0.84 cm^3^ g^−1^).^54^ Acetate capping compensates for the lower thermal stability of MOF-808, raising its decomposition temperature to 300 °C.^55^

In summary, all eight Zr-based MOFs discussed above can be synthesized *via* solvothermal methods;^56,57^ however, certain protocol customizations significantly reduce the reaction time (*e.g.*, PCN-224, MOF-525).^52,58,59^ With the exception of MIL-140(Zr), which contains ultramicropores smaller than 7 Å, most Zr-based MOFs exhibit relatively large pore sizes within the microporous to mesoporous range (Table 1). Such pore dimensions facilitate the diffusion and accommodation of small analyte molecules into the framework. The presence of mesopores enables the adsorption of some larger functional units, thereby broadening the potential applications of these MOFs. In addition, their high porosity facilitates the preconcentration of target analytes and provides abundant coordination sites for functional molecules. The high energy of Zr–O bonding (>760 kJ mol^−1^)^56^ results in stable frameworks with outstanding thermal stability^31,52^ and surprising robustness to acid conditions.^62^ These properties, uncommon among the different MOFs, position these Zr-based MOFs as suitable candidates for stable platform materials for diverse applications. We note that such stability is particularly important in a variety of applications, including biological, electrochemical, and sensing, where frameworks often need to withstand specific acid or base conditions.^62^

## Zr-based metal–organic frameworks for colorimetric sensing

Zr-based MOFs stand out for their structural designability, high porosity, and large surface area.^45,47,54,56^ This makes them a very special class of materials with promising applications in colorimetric sensing. The metal clusters, organic ligands, and porosity can be directly or indirectly used to interact with target analytes, with distinct colour changes. Therefore, Zr-based MOFs represent a good platform for the development of colorimetric sensors.

In this section, the colorimetric sensing applications of Zr-based MOFs are organized into four parts. The first one discusses their application in acid and base detection, including some acidic and basic vapours, especially those associated with proton transfer processes. The second part focuses on aqueous ion detection, covering metal ions, non-metal cations, and anions. The third part examines biomolecule detection, especially biogenic amines produced during meat spoilage, pesticide and veterinary drug residues, bacteria, glucose, amino acids, and peptides. The final part addresses the sensing of diverse gases and vapours.

### Acids and bases

The relationship between acidic and basic environments and colorimetric response is a recurrent aspect in chemistry and material science. For example, phenolphthalein and litmus are classical pH-responsive colorimetric sensors, in which acid–base induced molecular structural changes lead to visible colour transitions.^63^ Two principal approaches have been developed for constructing MOF-based colorimetric sensors: using pH-sensitive linkers (*e.g.*, porphyrin MOFs) or incorporating pH indicators into the framework.

Metal-free porphyrins are well-known to exhibit a distinct colour change from pink to green upon protonation in acidic conditions. This is attributed to the combination of several factors: (i) the change in symmetry from *D*_2h_ to *D*_4h_ upon conversion to the dication, resulting in degeneration of the 4 Q-bands in the visible part of the spectrum into two more intense bands^63,65^ and (ii) saddling distortion of the porphyrin macrocycle and adoption of the non-planar conformation due to steric crowding and electrostatic repulsion in the porphyrin dication.^63,66^

Similar to solutions in water or organic solvents, porphyrins undergo the same structural and spectral changes upon protonation when utilized as linkers in MOFs.

This phenomenon was first reported by Deibert *et al.*^62^ in 2014 as a distinct reversible colorimetric pH response on a Zr-based MOF. The authors found that the structure of PCN-222 can undergo a distinct and reversible colour change upon protonation, with the effect being particularly pronounced at low pH. At pH = 0 and pH = 1, the colour changes from purple to green, and turns back to purple after washing the MOF with DMF (Fig. 3). This study reveals that this colour change is not only attributed to the TCPP ligand. Indeed, when comparing the MOF system with the free ligand, at pH = 0, free TCPP molecules show only a colour change limited from purple to blue, and at pH 1, a colour change can hardly be detected by the naked eye. In contrast, the orderly arrangement of TCPP ligands within the framework enhances the optical properties of the TCPP molecules, thereby enabling PCN-222 to respond with a much greater colorimetric change under the same pH conditions. This work represents the first report of a MOF as a pH colorimetric sensor, where the visible colour change is attributed to protonation. It provides researchers with the evident advantage of a MOF crystal organising porphyrin ligand and Zr-based cluster in an accessible extended material for sensing applications.

Although a clear and distinct colour change is observed for porphyrin-based MOFs, such a process typically occurs below pH 5, making them suitable colorimetric sensors in acidic conditions.^67^ Moreover, as porphyrin rings are capable of chelating metal ions, these sensors are susceptible to metal coordination, even under mild conditions,^67^ which may lead to spurious positive responses in the presence of metal cation contaminants. Indeed, similar to protonation, chelation with metal ions results in a symmetry change from *D*_2h_ to *D*_4h_ and the appearance of two Q-bands instead of four.^65^

Stimulated by the aforementioned pioneering report, Smith *et al.*^68^ further quantified the colour change resulting from protonation of TCPP in MOF-545 (also known as PCN-222). The authors employed the International Commission on Illumination (CIE) L.a.b. Colour space^69^ to provide a rational analysis of the colour variation. This colour analysis system distinguishes colour by numerical changes along three coordinate axes, enabling the calculation of colour differences (Δ*E*) through the distance between coordinates. By measuring the colorimetric responses of MOF-545 to various concentrations and types of acids, the authors calculated the total colour change (Δ*E*) for each case. The results revealed that Δ*E* is influenced not only by proton concentration but also by the type of anions present. For example, when comparing the colorimetric response to HCl and citric acid, the Δ*E* value of citric acid at 0.5 M was comparable to that of HCl, and at 0.6 M it even exceeded the response of HCl. Moreover, among the various acids tested, phosphoric acid showed a higher Δ*E* than others. These data indicate that, within a specific pH range, MOF-545 can respond with different colorimetric changes to different acid types; the range is from brown to green. By using this property, the authors applied MOF-545 to detect four pesticides with different acidity levels (Fig. 4), finding that glyphosate produced the largest colour change among the tested insecticides.

In general, we note that MOF powders can be incorporated into various rigid and flexible matrices. Drawbacks range from sample contamination to sedimentation to limited recyclability, leading to unneeded reproducibility issues and superfluous costs. Therefore, such experiments should be regarded mainly as elegant proof-of-concept studies or preliminary investigations. For the sensor fabrication, it is desirable to implement a MOF positioning technology (*e.g.*, photolithography, imprinting, ink-jet printing^70^), minimizing readout variability and maximizing cyclability of the final sensing device.

Beyond PCN-222, similar protonation induced colorimetric responses have also been reported in other porphyrin ligand Zr-based MOFs. Sousaraei *et al.*^71^ reported that PCN-224 undergoes reversible protonation, accompanied by a distinct and reversible colour change from brown to green, readily observable to the naked eye (Fig. 5). Regeneration can be readily achieved by exposure to a base (Et_2_NH). The authors incorporated PCN-224 as a functional component into a highly porous polydimethylsiloxane (PDMS) matrix to fabricate mixed-matrix membranes (MMMs), which were applied to the detection of volatile alkaline gases such as Et_2_NH, Me_2_NH, and ammonia. In this composite, the optical properties of the pure MOF crystals are preserved. Additionally, compared to a MOF pellet, the polymer/MOF system affords a reduced amount of MOF material required to coat a given surface. The processability of polymers that can be crosslinked on demand (*i.e.*, temperature or light triggered polymerization^72^), and the retained performance of the MOF even under humid conditions can broaden the range of potential applications for the functional MOF.

Shifting focus to ionogel systems, MOFs have also been incorporated to enhance the mechanical and functional properties of the matrix. Yu *et al.*^73^ synthesized MOF-525 through solvothermal methods and employed it as a functional filler in a 3D printing ink to make a tough MOF-based ionogel (MIG). With the modification of MOF-based ionogels, even with a low MOF-525 loading of only 0.2 wt%, the MIG still retained the same purple colour observed on the MOF-525 powders. Interestingly, upon exposure to different acid conditions, the MIG displayed distinct colour changes: from pH 0 to 3, the colour change observed followed the light green, dark green, brown, and red sequence. However, when pH ≥ 4, no colour change could be observed by the naked eye. They successfully developed a wearable material with MIG by 3D printing, demonstrating a design strategy of multifunctional colorimetric sensors involving Zr-based MOFs (Fig. 6). This colorimetric sensor was thought to demonstrate the ability to detect acidic compounds, such as glyphosate pesticides, as sources of environmental pollution. However, considering that the sensor shows a colour change at pH < 4, which corresponds to a highly concentrated glyphosate solution, the system still has room for improvement, particularly in terms of sensitivity.

Post-synthetic modification is an important and widely used strategy to enhance the properties of MOFs or to introduce functional groups onto their frameworks,^74^ thereby expanding the application range of MOF-based colorimetric sensors. Kim *et al.*^75^ coordinated EDTA to the non-structural sites on the Zr_6_O_8_ metal clusters of MOF-808, and subsequently complexed metal ions (Cu^2+^ or Fe^3+^) with the post-synthetically modified MOF-808-EDTA to obtain MOF-808-EDTA-M (M = Cu or Fe). This Zr-based MOF composite material serves as a proton-triggered colorimetric sensor capable of detecting up to six acidic vapours (HCl, HI, HF, HBr, HNO_3_, and TFA) through a direct colour change observable by the naked eye (or analysable by camera-based RGB values). The colour change is partially reversible, with about 80% of Cu^2+^ ions re-chelated during the regeneration process, enabling the sensor to be reused multiple times. Notably, MOF-808-EDTA-M is not simply acid-triggered; for example, upon exposure to HCl, the EDTA ligands on the framework undergo protonation, leading to the loss of Cu^2+^ coordination. The released Cu^2+^ ions then bind to Cl^−^, resulting in a colour change from blue to greenish-yellow. Therefore, both Cl^−^ and H^+^ are essential for the colorimetric response of acidic vapours by MOF-808-EDTA-Cu (Fig. 7). Furthermore, by incorporating the flexible polymer polyvinylidene fluoride (PVDF), a portable miniaturized acid sensor can be fabricated.

Various device fabrication methods incorporate MOFs as functional fillers into devices.^70^ Among these approaches, the most common strategies include directly mixing MOFs with substrate materials or coating MOFs onto/with other functional materials, thereby expanding the application range.

Lee *et al.*^76^ developed a hybrid matrix air filtration material by incorporating a colorimetric multi-scale nanofiber (NF)/nanonet (NN) membrane onto a net substrate modified with UiO-66-NH_2_. The NN was fabricated by co-spinning bromocresol purple (BCP), a colorimetric dye, with a polyacrylonitrile solution. The integrated BCP on the composite material exhibited a rapid and reversible colorimetric response to ammonia gas concentrations as low as 20 ppm, with a colour change completed within 5 seconds. It should not be overlooked that it remains unclear whether the incorporation of the MOF was truly beneficial for NH_3_ sensing, as the colorimetric response originated from BCP, which was incorporated with the MOF into a filter matrix. Additionally, the porous UiO-66-NH_2_ further enhanced the efficient adsorption of NH_3_ and acetaldehyde, enabling effective air filtration.

Purple cabbage anthocyanins exhibit a colorimetric response to pH, with corresponding colours from pH 2 to 12, making them excellent pH colorimetric agents.^77^ However, anthocyanin molecules are often unstable and easily adhere to and stain other substances. Fang *et al.*^78^ prepared a composite matrix label material by mixing UiO-66-NH_2_, anthocyanins, and sodium alginate (SA). The cross-linked SA serves as the label substrate, providing a good platform. UiO-66-NH_2_ shields UV interference and enhances the interaction between anthocyanins and the label film substrate, resulting in the MOF-anthocyanin label exhibiting good colorimetric responsiveness to pH (Fig. 8). The material exhibits high sensitivity and rapid response to NH_3_, dimethylamine (DMA), and trimethylamine (TMA), while remaining unaffected by interference from alcohols, aldehydes, and gaseous sulfides, making it suitable for visually indicating early spoilage in shrimp.

The high porosity of MOF structures allows functional dyes to be adsorbed onto their internal surfaces. Du *et al.*^79^ loaded alizarin complexone, a colorimetric pH-responsive material, onto the acid- and alkaline-stable fluorescent MOF, UiO-66-NH_2_, to create the composite material AC@MOF. AC@MOF exhibited a colorimetric response to pH, exhibiting a significant colour change from light yellow to dark pink. In 2025, the same research group combined AC@MOF with a flexible substrate composed of sodium alginate (SA) and bacterial nanofibrillar cellulose (BNC) to create a smart tag based on SA/BNC-AC@MOF.^80^ This tag exhibited a highly sensitive colorimetric response to pH changes, with colour changes ranging from light yellow to deep red depending on pH value. Furthermore, the fluorescence change under UV irradiation was visible to the naked eye, ranging from light blue to purple. Experiments demonstrated a strong linear relationship (*R*^2^ = 0.991) between RGB colorimetric intensity and pH.

Using a similar strategy, Aham *et al.*^81^ prepared UiO-66-NH_2_@RhB by shaking Rhodamine B (RhB) with UiO-66-NH_2_ for use as a colorimetric sensor to detect perfluorooctanesulfonate (PFOS), a man-made fluorosurfactant now regarded as a persistent acidic pollutant. The coordination between PFOS and RhB triggered structural changes, causing a ring-open reaction of RhB and a rapid fading of the solution's red colour. The material achieved a detection limit of 0.91 nM in real water samples.

Due to their excellent application stability and designable structure, Zr-based MOFs have been widely used as colorimetric sensors for real-time, rapid, and highly sensitive pH monitoring.^75,76^ Particularly, MOFs constructed by the TCPP ligand, such as PCN-222,^62,68^ PCN-224^70^ and MOF-525,^73^ show reversible colour change in response to pH variations, demonstrating great potential for practical applications.

### Aqueous ions

Ionic pollution in water is a global environmental problem.^82,83^ Conventional technologies for the identification and/or removal of ions from solutions, especially from aqueous media, are often lengthy, costly, and cumbersome processes. In contrast, the large capacity of porous MOFs offers a promising platform for both ion detection and adsorption, enabling their removal in a more cost-effective and efficient manner.^84,85^

**Fig. 2:**
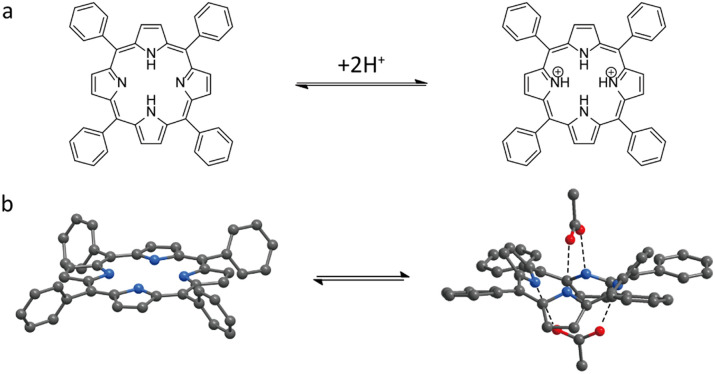
(a) Reversible structural changes of porphyrins upon protonation and deprotonation and (b) saddling distortion of the porphyrin macrocycle and adoption of the non-planar conformation.^63–66^

As noted in the previous section of this review, the structure of porphyrin (Fig. 2a) can be altered through protonation and deprotonation processes.^86^ In addition to protons, metal cations can also coordinate with the central cavity of the porphyrin ligands in MOFs, thereby inducing structural changes in the framework.^87,88^ Similar to the protonation process in porphyrin MOFs, the incorporation of metal ions results in the symmetry change to *D*_4h_, which can lead to distinct colour changes in the framework. Yang *et al.*^89^ employed porphyrin ligands in combination with Hg^2+^ to develop a PCN-224 framework, synthesized by a solvothermal method in HEPES buffer solution (60 mM, pH = 7), as a colorimetric probe for the selective detection of Hg^2+^ in aqueous solution. In this system, the porphyrin ligand within PCN-224 framework acts as the recognition site, selectively coordinating Hg^2+^ at concentrations as low as 0.1 μM in mixed cation solutions, accompanied by a distinct colour change from purple to light green within 2 minutes (Fig. 9). Moreover, the colorimetric probe can be regenerated through treatment with KI solution, although the use of a particle dispersion is not the most convenient set-up for a sensor. This work suggested that incorporating metal ions into the core of porphyrin ligands in MOF structure could be extended to the convenient colorimetric detection of a broader range of metal ions. On the other hand, it should not be overlooked that the materials for pH sensing discussed above, especially those porphyrin MOFs, may also exhibit cross-sensitivity toward heavy metal ions, which could lead to false positive results if not carefully controlled.^67^

**Fig. 3:**
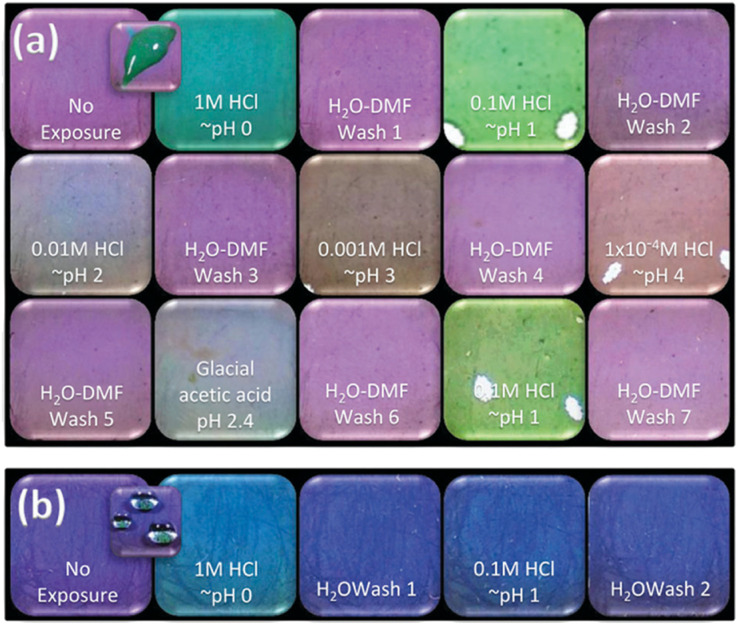
Photographs of PCN-222 samples (a) and ligand TCPP (also called H6tcpp) (b) under ambient light. These samples are exposed to acidic solutions at various concentrations, followed by reversal washes in between each exposure; exposures were made in the order in which they appear from top left to bottom right. Caption photos are intended to demonstrate responsiveness. Reproduced from ref. 62 with permission from the Royal Society of Chemistry, Deibert *et al.*, *Chem. Commun.*, 2014, **50**, 9636, Copyright 2014.

**Fig. 4:**
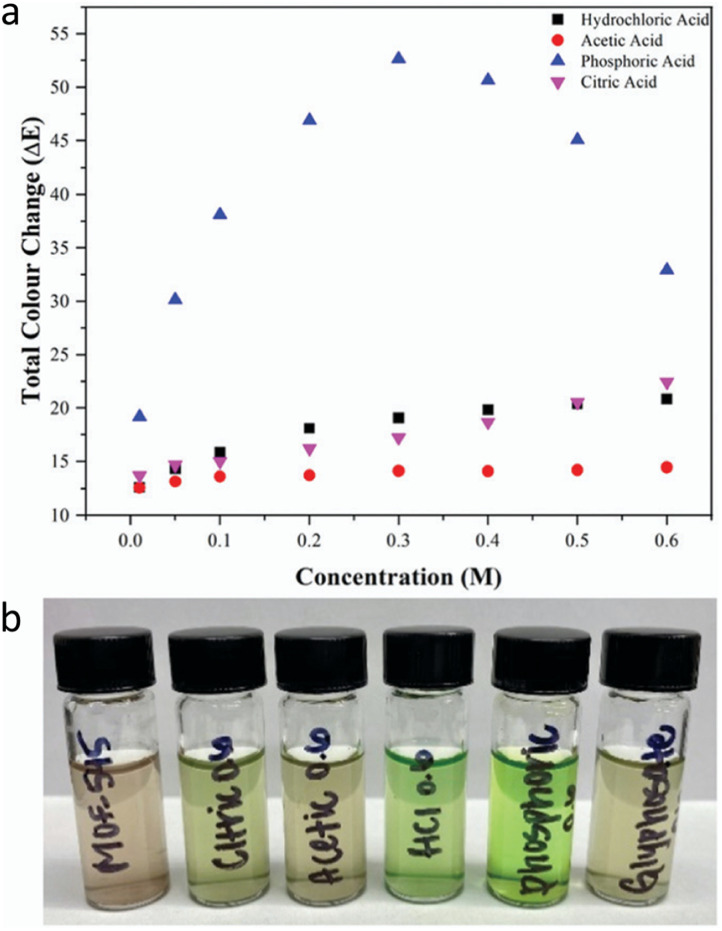
(a) Colour response to varying concentration of acid solutions and (b) the acid solutions after the addition of MOF-545. Reproduced from ref. 68 with permission from the Royal Society of Chemistry, Smith *et al.*, *Chem. Commun.*, 2022, **58**, 953, Copyright 2022.

**Fig. 5:**
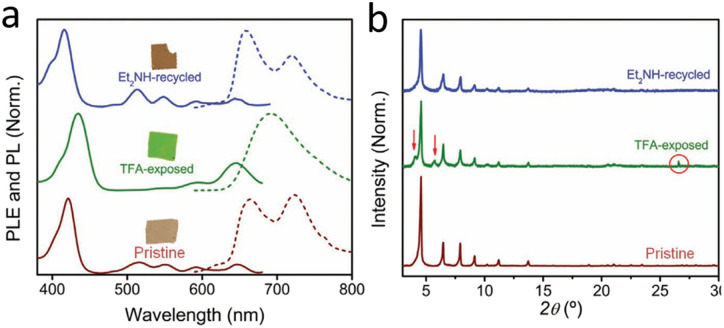
(a) The absorption (and fluorescence spectra) of PCN-224 MMMs with distinct and reversible colour change of PCN-224 MMMs in different atmospheres; (b) PXRD patterns confirming reversible structural transformations during the colour transition. Reproduced from ref. 71 with permission from Wiley-VCH GmbH, Sousaraei *et al.*, *Adv. Mater. Interfaces*, 2021, **8**, 2001759, Copyright 2021.

**Fig. 6:**
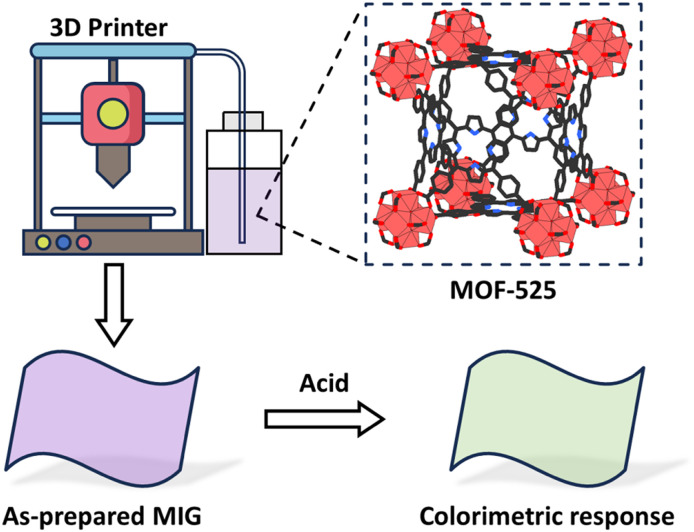
Design of a 3D-printable MOF-based ionogel (MIG). Scheme inspired by ref. 73, Yu *et al.*, *ACS Appl. Mater. Interfaces*, 2023, **15**, 39319–39331.

**Fig. 7:**
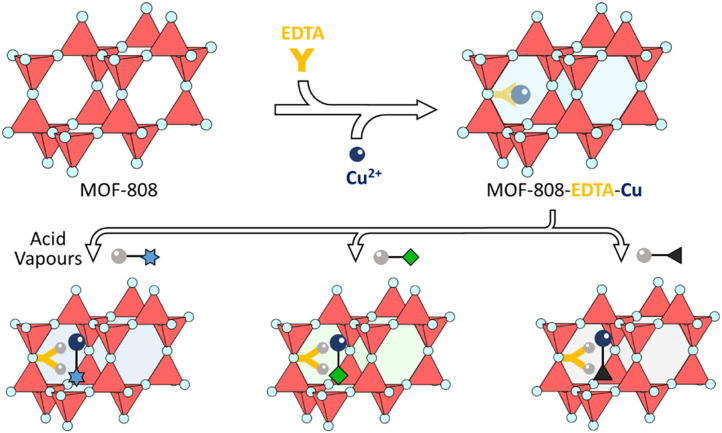
Schematic illustration of the preparation of MOF-808-EDTA-Cu and its application as a colorimetric acid vapor sensor. Scheme inspired by ref. 75, Kim *et al.*, *Nat. Commun.*, 2025, **16**, 385.

**Fig. 8:**
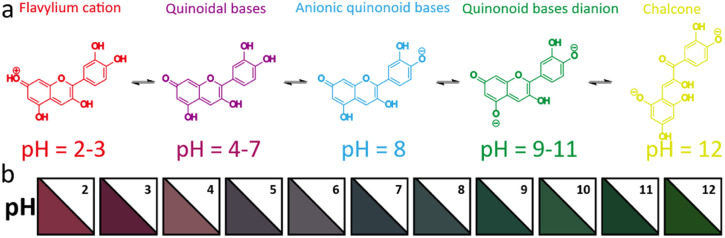
(a) UV-vis spectral changes of red cabbage anthocyanin at a pH range from 2 to 12, (b) UiO-66-NH_2_ composite matrix label material as a colorimetric sensor for monitoring pH over the range of 2–12. Scheme inspired by ref. 78. Fang *et al.*, *Int. J. Biol. Macromol.*, 2024, **276**, 133914.

**Fig. 9:**
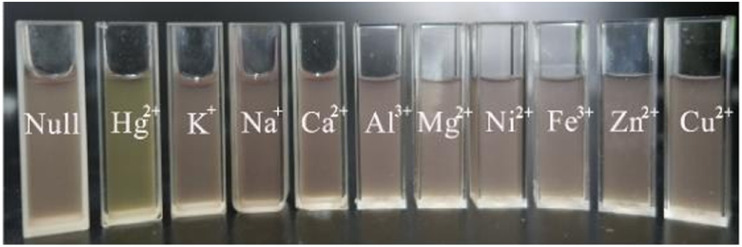
The colorimetric change of PCN-224 in the presence of other metallic cations (5 μM) and Hg^2+^ (5 μM). Reproduced from ref. 89, with permission from the Royal Society of Chemistry, Yang *et al.*, *RSC Adv.*, 2016, **6**, 69807–69814, Copyright 2016.

**Fig. 10:**
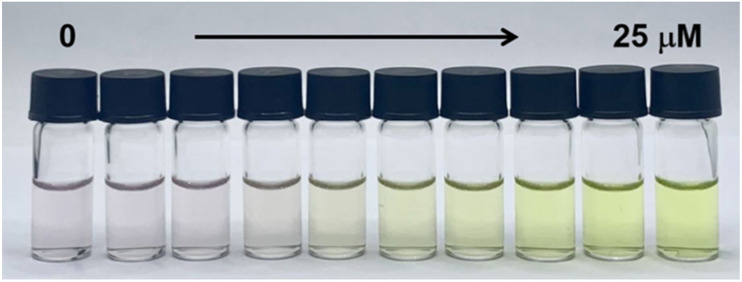
The colorimetric response of PCN-222 + PO_4_^3−^ to different concentrations of Pb^2+^. Reproduced from ref. 90 with permission from Elsevier, Liu *et al.*, *Sens. Actuators, B*, 2024, **420**, 136455, Copyright 2024.

**Fig. 11:**
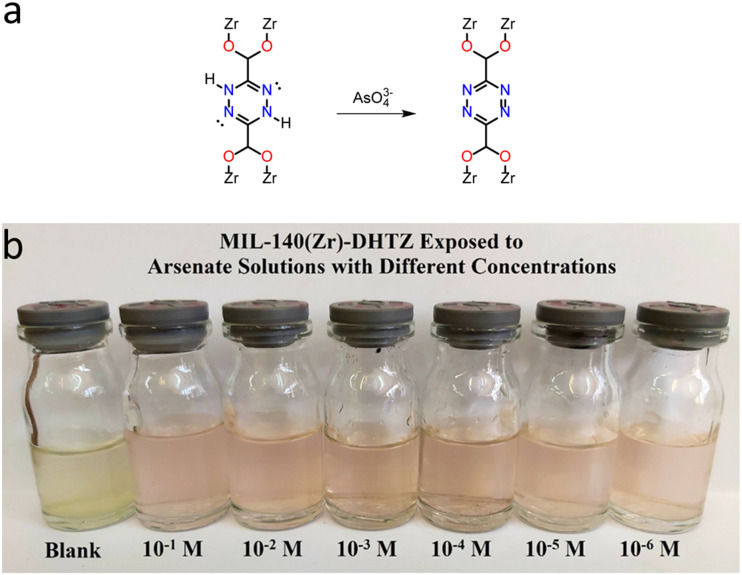
(a) Change of MIL-140(Zr)-DHTZ upon exposure to arsenate, (b) colorimetric response to varying concentrations of arsenate. Reproduced from ref. 93 with permission from the American Chemical Society, Razavi *et al.*, *ACS Appl. Mater. Interfaces*, 2023, **15**, 39319–39331, Copyright 2023.

**Fig. 12:**
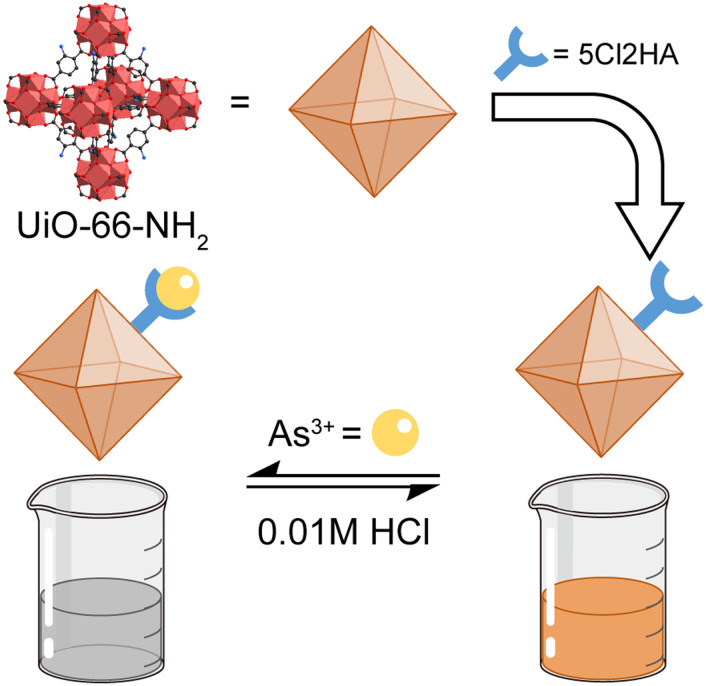
Schematic illustration of the preparation and colorimetric sensing process of 5Cl2HA=(Zr) MOF. Scheme inspired by ref. 95, Alshammari *et al.*, *J. Mol. Liq.*, 2023, **389**, 122787.

**Fig. 13:**
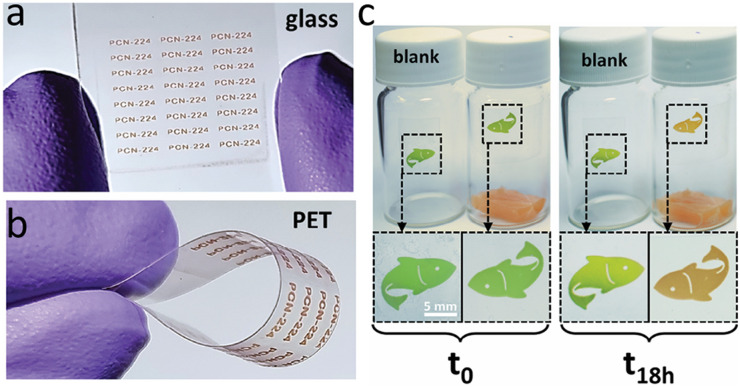
The colorimetric label fabricated by printing MOF-ink containing PCN-224 on (a) glass and (b) PET substrates, (c) exhibit a colour change from green to orange after 18 hours of exposure to fresh fish samples. Reproduced from ref. 109, Carbonell *et al.*, *Adv. Mater.*, 2024, **36**, 2408770, licensed under CC BY 4.0.

**Fig. 14:**
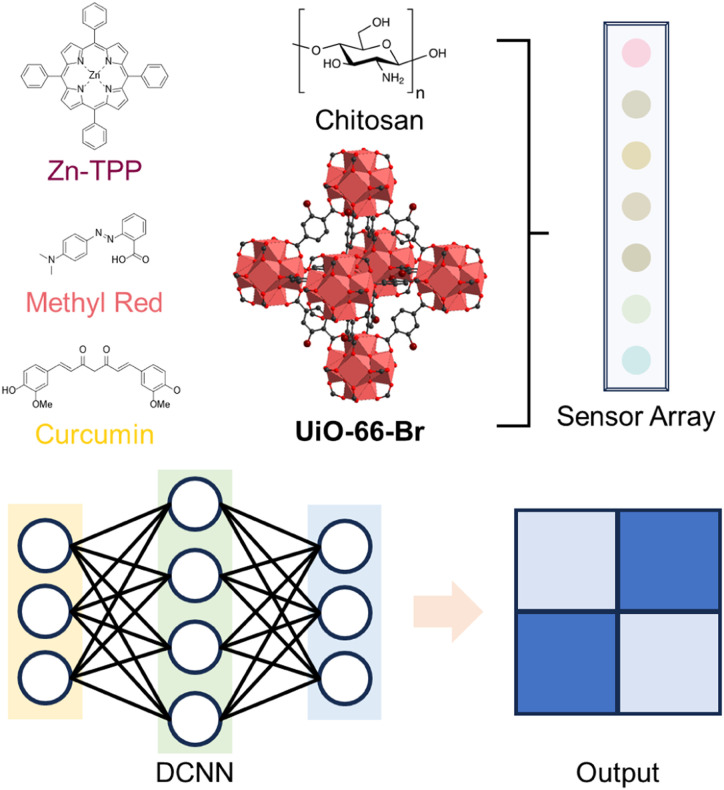
Fabrication of dye@MOF colorimetric sensor array and its application with a deep convolutional neural network (DCNN). Adapted from ref. 123 with permission from the American Chemical Society, Ma *et al.*, *ACS Sustainable Chem. Eng.*, 2021, **9**, 16926–16936, Copyright 2021.

**Fig. 15:**
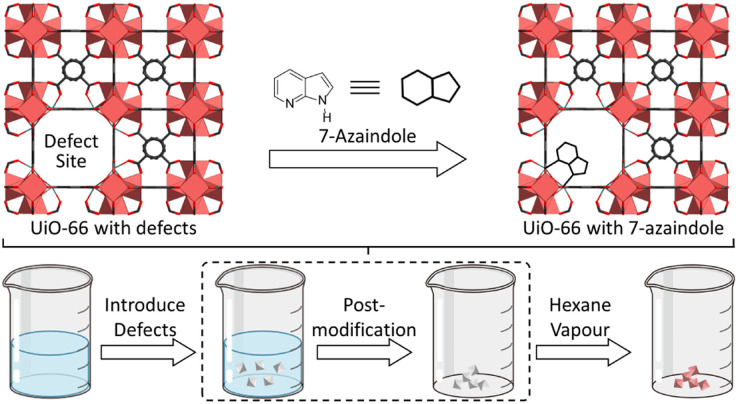
A colorimetric alkane recognition system composed of UiO-66 with defects and 7-azaindole. Adapted from ref. 133 with permission from the American Chemical Society, Takashima *et al.*, *Inorg. Chem.*, 2016, **55**, 11617–11620, Copyright 2016.

**Fig. 16:**
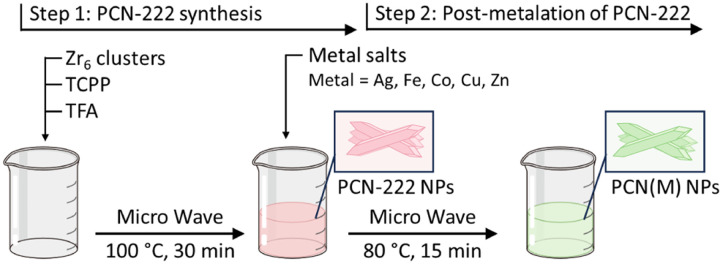
The preparation of PCN-222 and PCN(M). Adapted from ref. 137, Moscoso *et al.*, *Small Sci.*, 2024, **4**, 2400210, licensed under CC BY 4.0.

**Fig. 17:**
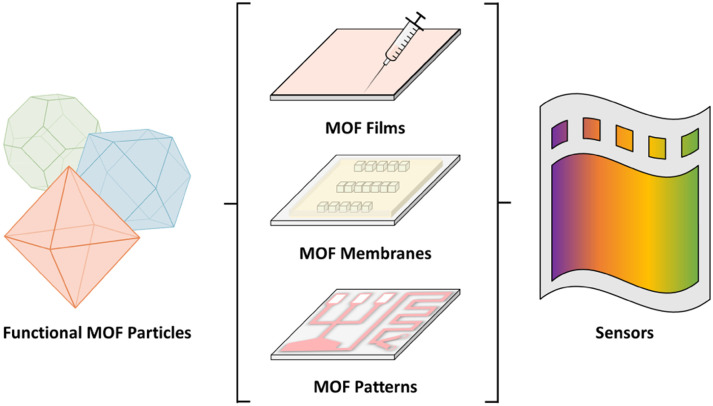
From MOF particles with sensing properties (probes) to sensors *via* fabrication of MOF films, membranes, and patterns.

Subsequently, Liu *et al.*^90^ developed a “traffic light”-type colorimetric assay for the selective detection of heavy metal ions (Cd^2+^ and Pb^2+^) in city water samples. This ion-responsive colorimetric system, denoted as PCN-222 + PO_4_^3−^, appeared as a light red solution and was prepared by mixing PCN-222, synthesized by a solvothermal method, with sodium phosphate and 10 mM Tris-HCl. In the presence of Cd^2+^ in tap water, the light red solution changed to green, whereas the presence of Pb^2+^ caused a colour change to yellow (Fig. 10). These colour changes come from interactions between the metal ions and porphyrin ligands. Furthermore, after binding with heavy metal ions, the PCN-222 + PO_4_^3−^ complexes could be separated by centrifugation, enabling water purification.

Following a similar strategy of using MOF ligands as coordination sites for target analytes, Wang *et al.*^91^ incorporated a chromophoric ruthenium complex (Ru(H_2_bpydc)(bpy)(SCN)_2_) into UiO-67, developing a so-called RuUiO-67, a specific and reversible colorimetric sensor for Hg^2+^ with a distinct colour change from red to yellow due to coordination of the metal ion to the thiocyanate ligands. The two SCN (thiocyanate) groups of the Ru complex coordinate with Hg^2+^ in water, enabling detection at concentrations as low as 12 μM. RuUiO-67 exhibited high selectivity toward Hg^2+^ even in the presence of other competing cations, and the probe could be regenerated by treatment with I^−^.

Because of their designable structures, MOFs are often subjected to post-synthetically modifications to impart additional unique functions to otherwise stable frameworks.^74^ Through these modifications, a wide variety of functional units can be incorporated into the framework, thereby extending their utility far beyond that of simple functional molecules.

Building on the concept of post-synthetic functionalization to impart specific sensing properties, Khalil *et al.*^92^ treated the pore surfaces of UiO-66 with *N*-dodecylpyridinium chloride (NDC) as a cationic detergent to impart a positive charge to the framework. The UiO-66 pores were then functionalized with sodium diethyldithiocarbamate (DDTC) to obtain DDTC/UiO-66, which exhibited a rapid colorimetric response to Cu^2+^ ion in water, changing from white to yellow. The colour change was caused by the complexation of Cu^2+^ with DDTC, and the yellow colour intensified with increasing Cu^2+^ concentration. RGB analysis further revealed that the intensity of the sensor in the blue channel decreased as the Cu^2+^ concentration increased.

Taking advantage of the one-dimensional channels in a water-stable Zr-based MOF MIL-140(Zr) (also known as MIL-140-A), Razavi *et al.*^93^ employed it as a template and replaced the BDC ligands in MIL-140(Zr) with H_2_DHTDCA (dihydro-1,2,4,5-tetrazine-3,6-dicarboxylic acid) by a secondary solvothermal method to obtain MIL-140(Zr)-DHTZ. Because the DHTZ ligand shares a similar *para*-position dicarboxylic acid structure in its six-membered ring with BDC, MIL-140(Zr)-DHTZ retained the same structure as MIL-140(Zr). In the presence of arsenate (AsO_4_^3−^) in aqueous solution, the suspension of MIL-140(Zr)-DHTZ displayed a distinct colour change from yellow to pink (Fig. 11), caused by the chemical transformation of the DHTZ ring to a tetrazine ring. MIL-140(Zr)-DHTZ possesses a highly sensitive colorimetric response, enabling detection of arsenate at concentrations as low as 1 nM in water samples.

Beyond arsenate (AsO_4_^3−^), arsenite (As^3+^) is another hazardous pollutant of concern in municipal water environments.^94^ Alshammari *et al.*^95^ condensed 5-chloro-2-hydroxyacetophenone onto the amino groups of the UiO-66-NH_2_ MOF, obtaining a post-functionalized framework named 5Cl2HA=(Zr) MOF. 5Cl2HA=(Zr) exhibited a rapid spectrophotometric response to As^3+^, detecting concentrations as low as 0.0607 ppm in water samples within 10 seconds (Fig. 12). The solution colour changed from light orange-yellow to progressively lighter shades and eventually became nearly colourless as the As^3+^ concentration increased. In addition, 5Cl2HA=(Zr) MOF could be regenerated by washing with HCl.

Using a similar strategy, Motora *et al.*^96^ employed a diazo coupling reaction to introduce the azo functional groups, obtaining UiO-66-Azo-Im. The post-synthetically modified framework was grafted onto a carboxymethyl cellulose film to form a UiO-66-Azo-Im@CCM composite sensing material, which exhibited a sensitive colorimetric response to heavy metal ions Cu^2+^ and Co^2+^ in water, with a distinct colour change from yellow to orange-red (Cu^2+^) and orange (Co^2+^). The Cu^2+^ and Co^2+^ were adsorbed inside the framework through hydrogen bonding interactions with the amino groups of the ligands, causing energy and charge transfer between the framework and metal ions, thereby leading to the observed colour change.

In addition to the internal surfaces and pores of MOF structures, the external surfaces of MOF particles, which often exhibit a regular polyhedral shape, can also be utilized for post-synthetic modification to introduce functional units.^97^

Building on this approach, Li *et al.*^98^ developed a colorimetric sensor by loading the fluorescent dye FITC (fluorescein isothiocyanate) onto UiO-66-NH_2_ by simply mixing in water, enabling selective detection of F^−^ ions in aqueous solutions. UiO-66 loaded with FITC exhibited a distinct colour change from colourless to yellow when the F^−^ concentration exceeded 0.9 ppm. This colour change was attributed to the capture of F^−^ ions by the amino groups of the framework, which subsequently affected the interaction between FITC and F^−^.

Beyond fluoride ions, nitrite (NO_2_^−^), an important nitrogen-containing inorganic ion extensively employed in food processing, attracted more and more attention because of its widespread occurrence in food, water, and biological samples.^99^ In 2025, Fu *et al.*^100^ covalently immobilized horseradish peroxidase (HRP) on the amino groups of UiO-66-NH_2_. The high surface area and porosity of UiO-66-NH_2_ enhanced enzyme immobilization and substrate mass transfer, and HRP catalyzed TMB/H_2_O_2_ oxidation. In the presence of NO_2_^−^, oxTMB undergoes a diazotization reaction, providing a visible colour change from blue to green to yellow, enabling a detection range of 5–250 μM.

In the same year, they further fabricated the HRP-on-UiO-66-NH_2_ into a portable test kit made with gelatine, extending the upper limit of detection up to 400 μM.^101^ Although HRP is introduced in these two nitrite sensors, its role is primarily catalytic rather than biological recognition. Enzyme-MOF composites have also been widely employed in biomolecule sensing, as illustrated in the next subsection.

MOFs offer multiple coordination sites for metal ions through both their ligands and SBUs, a feature particularly evident in PCN-222^90^ and PCN-224,^89^ which are constructed by porphyrin ligands. Meanwhile, robust Zr-based frameworks can be functionalized, further expanding the available coordination sites for metal ions,^91,92,96^ non-metal cations,^95^ and anions^93,98,100,101^ in aqueous solutions. The development of MOF colorimetric ionic sensors offers an affordable and easily controllable approach for real-time water quality monitoring, which is especially important in regions facing water scarcity.

### Biomolecules

Biomass sensing is of great importance in food and hygiene fields, as it enables rapid detection and monitoring of microbial biomass, thereby ensuring food safety, preventing contamination, and supporting public health protection.^102–104^

A key indicator of food spoilage is the presence of biogenic amines (BAs),^105,106^ and real-time, rapid detection is essential for maintaining food quality and safety.^107,108^ Carbonell *et al.*^109^ fabricated a colorimetric label by printing a MOF-ink containing PCN-224 through digital-light processing (DLP) for the reversible colorimetric response to biogenic amines released during fish spoilage. The printed MOF label exhibited a colour change from green to orange after 18 hours of exposure to fresh fish samples. Unlike the common solvothermal method (Fig. 13), the authors first synthesized Zr_6_ metal clusters and subsequently stirred them with TCPP ligands in the solvents at room temperature to obtain PCN-224 as a purple powder. After treatment with HCl, the powder turned green because of protonation of the porphyrin ligands in PCN-224, with the purple-to-green colour change^62^ discussed in the “acids and bases” section of this review. Exposure to biogenic amines triggered the reverse deprotonation process, which led to the observed colour transition. This strategy provides an efficient and convenient approach for real-time monitoring of food freshness.

Thanks to the high stability of Zr-based MOFs, functional modifications allow the integration of different properties into a single material, achieving a synergistic effect.^74^

Metal nanoparticles, especially gold nanoparticles (Au NPs), are widely used to enhance the colorimetric signal because of the localized surface plasmon resonance (LSPR) effect.^110,111^ Wen *et al.*^112^ integrated photoactive Au nanoparticles (NPs) onto UiO-66, yielding UiO-66/Au NPs with enhanced hydrolysis activity under light excitation. Under illumination at 652 nm, the material hydrolysed ethyl-paraoxon (a pesticide metabolite that can contaminate food) to *p*-nitrophenol producing an increasingly intense yellow colorimetric response. Because the Zr_6_ clusters in UiO-66 resemble the catalytically active sites of natural phosphatases, the paraoxon adsorbed within the pores can coordinate with these metal clusters. The local catalytic microenvironment temperature of UiO-66/Au NPs increased under illumination, thereby enhancing the affinity between the Zr_6_O_8_ metal clusters within UiO-66/Au NPs and the hydrolytic substrate. This material combines rapid colorimetric detection and ethyl-paraoxon degradation, providing an effective approach to organophosphorus compounds monitoring and remediation.

Because of the designable structure of MOFs, core–shell MOF composite materials with diverse designability and enhanced selectivity have inspired high scientific interest.^113^ This strategy synergistically combines the advantages of both components, broadening their applicability across diverse conditions.^114^ Inspired by this strategy, Hui *et al.*^115^ developed a novel core–shell material (UiO@TBTA) with a colorimetric response to glutathione (GSH) by *in situ* growth of TFPB-TAPA COF (TFPB = 1,3,5-tris[4-formylphenyl]benzene, TAPA = tris[4-aminophenyl]amine) on the surface of UiO-66-NH_2_ through Schiff base reaction. This MOF@COF composite was applied for colorimetric sensing of GSH in human serum and blood samples, exhibiting a clear colour change from orange to black, which was attributed to hydrogen-bond formation between GSH and the MOF@COF sensor. In addition, intelligent artificial neural network models were employed to achieve precise quantification (1–200 μM) of GSH levels.

Owing to the good stability of Zr-based MOFs, employing them as substrates to expand the application range of biomimetic materials and antibody systems, which can provide specific detection of target analytes, has become a popular strategy.^116,117^

Building on this strategy, Shang *et al.*^118^ developed a biosensor based on bimetallic nanoparticles integrated with a MOF (AgPt/PCN-223-Fe). By modifying the material with anti-*E. coli* O157:H7 antibodies, Ab2@AgPt/PCN-223-Fe was prepared for colorimetric detection of *E. coli* O157:H7 in the range of 10^3^–10^8^ CFU mL^−1^, with a visible colour change from colourless to blue-green. In addition, AgPt/PCN-223-Fe exhibited an antibacterial rate of 99.94% against *E. coli* O157:H7 in the presence of small amounts of H_2_O_2_ with near-infrared (NIR) irradiation for 20 minutes. The assay was conducted in a portable 3D cassette, and the results were analysed using a smartphone equipped with a customized application.

In 2024, Zhong *et al.*^119^ employed PCN-222 as a nanotag conjugated with antibodies to prepare PCN-222–mAbs, which exhibited optical properties and a colorimetric response toward 3-[(4-carboxyphenyl)monomethyl] amino-2-oxazolidinone (CPAOZ), a derivative of furazolidone metabolite, as common veterinary drug residues found in food. The paper-based PCN-222-lateral flow assay was successfully applied to detect CPAOZ in shrimp, chicken, and milk samples.

Building on these lateral flow assays, Ren *et al.*^120^ employed UiO-66-NH_2_ as a high surface area scaffold to stabilize and disperse Pt nanoparticles (PtNPs) with high molar absorption coefficients, improving signal intensity and background tolerance in a dual-site immunochromatographic assay for the detection of ZEN (zearalenone) and FB (fumonisin B), two hazardous food contaminants commonly found in corn. However, in this LFIA (lateral flow immunoassay) system, the colorimetric response originates from the PtNPs rather than the MOF itself, which conceptually differs from MOF-intrinsic colorimetric sensing.

Huang *et al.*^121^ applied a core–shell approach in which PCN-222 was coated with liposomes, enabling its use as a stable platform for an LFIA method. The paper-based PCN-222@liposomes-LFIA sensor was applied to detect sulfonamides (SAs), a class of veterinary drugs, in chicken, shrimp, and milk.

The judicious design of a sensor array can overcome the limitations in sensitivity and clarity of a single sensor.^122^ Ma *et al.*^123^ developed a paper-based cross-reactive colorimetric sensor array, termed dye@UiO-66-Br/chitosan, by combining dyes with MOF particles (UiO-66 and UiO-66-Br) and integrating with a deep convolutional neural network (DCNN) platform (Fig. 14). Seven selected dyes, each responsive to specific biogenic amines or pH ranges, exhibited distinct colour changes. By analysing the scent fingerprint generated from the colorimetric responses of multiple dye@MOF functional units to volatile biogenic amines released during shrimp spoilage, the DCNN could accurately assess shrimp freshness. The integration of artificial intelligence (AI) with MOF-based sensors shows promising potential for future applications.

With a similar strategy, Zhao *et al.*^124^ developed a carboxymethyl cellulose-based colorimetric nano-sensor array incorporating ionic liquid-tuned anthocyanins, UiO-66, and nano-silica for real-time monitoring of pork freshness. The integration of UiO-66 significantly improved the sensor's mechanical properties, thermal stability, and sensitivity. The colorimetric unit that relies on four ionic liquid-tuned anthocyanins detected five volatile amines released during pork spoilage. Predictions of pork freshness using four deep convolutional neural network models all achieved accuracy rates above 97%.

Owing to the high substrate specificity of enzymes, enzyme-MOF composites have been widely applied in biomolecule sensing, especially for glucose, amino acids, and other small biomolecules.^125–128^ Within these composites, MOFs typically provide protection and enable the enzyme to maintain its activity in the presence of physical, chemical, and mechanical stressors.^127^ By adopting this approach, Ilacas *et al.*^129^ immobilized glucose oxidase (GOx) onto the surface of the PCN-222(Fe) framework by simply mixing it in the solvent to obtain a GOx-on-MOF system. However, it has been clearly reported that the notation enzyme@MOF should be used when the enzyme is encapsulated or infiltrated within the MOF framework, whereas enzyme-on-MOF is appropriate when the enzyme is immobilized on the outer surface of the MOF particles^127^ (this nomenclature will be used in the current review paper). The resulting GOx-on-MOF was further encapsulated with multiple layers and printed onto chromatography paper to fabricate a paper-based microfluidic device for the colorimetric detection of glucose. KI solution and the test sample were added to the designed holes on the paper-based assay, where the presence of glucose in the sample induced a colour change from colourless to yellow and then brown with increasing glucose concentration. The average inverse yellow intensity of the paper-based sensor showed positive correlation with glucose concentration.

Another similar study by Zhong *et al.*^130^ reported the selective colorimetric detection of glucose using the same GOx-on-MOF-type materials. They mixed GOx-on-MOF-545(Fe)(equal to PCN-222(Fe)) with ABTS (2,2′-azino-bis(3-ethylbenzothiazoline-6-sulfonic acid)) in PBS buffer, which catalysed glucose through a catalytic cascade reaction, exhibiting excellent ability to detect low glucose concentrations (0.28 μM) with high specificity. In various saccharide aqueous solutions with fructose, maltose, lactose, sucrose, and glucose, only glucose induced a colorimetric change from colourless to blue-green. With the protection offered by the MOF-545(Fe) surface, the recyclability and long-term stability of immobilized GOx were substantially improved when compared to free GOx.

With a similar approach, Li *et al.*^131^ immobilized GOx onto hierarchically porous MOFs (HP-MOFs) to achieve multi-enzymatic cascade reactions, enabling a colorimetric response to glucose. They synthesized HP-PCN-222(Fe) with different mesopore distributions by using a modulator-induced-defect formation strategy. HP-PCN-222(Fe) facilitated cascade reaction efficiency for glucose colorimetric detection by catalysing the oxidation of the 3,3′,5,5′-tetramethylbenzidine (TMB), which makes a colourless solution turn light yellow with limit of detection (LOD) as low as 20 μM.

Besides GOx, the previously mentioned horseradish peroxidase (HRP) is another commonly used enzyme; Dong *et al.*^132^ employed a bimetallic Ce/Zr-UiO-66 MOF with high surface area and structural stability as a platform to immobilize HRP for colorimetric signal generation and a BSA–chloramphenicol (bovine serum albumin–chloramphenicol) conjugate as a competing antigen in an immunosensing system. Chloramphenicol in water, fish, and urine samples was quantified based on a competitive immunoassay, exhibiting a concentration-dependent colorimetric response from deep blue to pale blue. In addition, the Ce (Ce^3+^/Ce^4+^ pairs) within the MOF enhanced the HRP catalyzed oxidation of TMB, leading to an accelerated colorimetric response.

With defined chemical and crystalline structures, researchers can design MOF composite materials based on selected structures of frameworks for specific target bioanalytes.^109,112^ However, post-synthetic modification method remains a popular strategy, allowing the functional molecules to be incorporated and achieving improved properties with simple fabrication approaches such as paper-based platforms,^118–121,123,129,132^ casting methods,^124^ sensor assays,^123^ and solution processing.^115,130,131^ In general, MOF-based colorimetric biosensors offer a versatile platform to assess food quality and hygiene conditions, with the potential to improve people's life quality and environmental sustainability.

### Volatile and gaseous analytes

By introducing defects into the well-defined structure of UiO-66, Takashima *et al.*^133^ reported an alkane recognition system based on a combination of UiO-66 and 7-azaindole. The authors synthesized UiO-66 by adding aqueous HCl, which protonated BDC ligands and prevented the complete coordination of Zr_6_O_8_ metal clusters, thereby introducing defects within the framework. Subsequently, 7-azaindole could be induced by hydrophobic alkane solvents to enter the pores of UiO-66 and coordinate to the defect sites, accompanied by a distinct colorimetric change from white powder to red (Fig. 15). This colorimetric change was significantly enhanced with increasing the defect concentration in the framework. Although Takashima *et al.* proposed a strategy for designing a MOF-based sensor capable of detecting alkanes, the quantitative assessment of the LOD of the current system has not yet been reported.

Isoreticular expansion^134^ allows changing the metrics of the linker while retaining the overall topology. By replacing the original BDC ligands with pre-modified 2,5-dinitro-1,4-benzenedicarboxylic acid (H_2_BDC-(NO_2_)_2_) ligands, Nandi *et al.*^135^ synthesized UiO-66-(NO_2_)_2_, an isostructural analogue of UiO-66, through a conventional solvothermal method. This material exhibited a selective colorimetric response to H_2_S in a simulated biological medium (HEPES buffer, pH = 7.4), with the solution colour changing from pale yellow to a distinct red. The authors also found that UiO-66-(NO_2_)_2_ could exclude other interfering compounds in the buffer matrix, demonstrating a highly selective colorimetric response to H_2_S with a detection limit as low as 14.14 μM. This material is likely to be applicable to a variety of detection scenarios, ranging from human plasma up to municipal water samples.

Using the open sites on the Zr_6_ clusters not participating in framework formation, Marquaedt *et al.*^136^ post-synthetically coordinated Cu(ii), introduced as CuCl_2_ or Cu(OAc)_2_, onto the Zr_6_ clusters of MOF-808 to synthesize Cu-MOF-808. The incorporated copper ions in Cu-MOF-808 showed a colour change from light green to light blue during activation, and a rapid shift to brown upon exposure to H_2_S gas. This distinct colour change is due to a redox reaction between H_2_S and Cu(ii) within the pores, temporarily reducing Cu(ii) to Cu(i). A cyclic test demonstrated that Cu-MOF-808 undergoes rapid reversible colorimetric response to H_2_S vapour at a concentration as low as 100 ppm within 2.2 minutes, with regeneration achieved by heating and cooling under humid conditions.

Building on the previously described mechanism of cations' interaction with porphyrin ligands, Moscoso *et al.*^137^ developed a MOF-based sensor array for colorimetric detection of various gases and vapours. The colorimetric sensor matrix was constructed from PCN-222 and PCN-222 variants with 5 metals in the central cavity (Ag^+^, Zn^2+^, Fe^2+^, Cu^2+^, and Co^2+^) *via* a microwave-assisted synthesis method (Fig. 16). Subsequently, MOFs were incorporated into a PDMS polymer to prepare PCN@PDMS membranes. In total, 12 test volatile and gaseous analytes were examined, including acidic vapor, basic vapor, nitroaromatic compounds, volatile organic compounds, polar and nonpolar solvent vapor. Each PCN@PDMS exhibited distinct colorimetric intensities and response patterns toward different analytes, enabling discrimination through the overall matrix. For instance, PCN-222@PDMS displayed a rapid and strong colour change from brown to green within 4 seconds upon exposure to HCl vapor, but showed no response to tetrahydrofuran (THF) vapor. The sensor matrix demonstrated the capability for distinguishing gases and vapours with different functional groups and acid–base properties through barcode-like recognition patterns, while also exhibiting high stability and maintaining performance after storage for over three months.

With the assistance of AI, You *et al.*^138^ developed an olfactory visualization assay combining a colorimetric array with densely connected convolutional networks (DenseNet) for detecting ethylene. UiO-66-NH_2_ was used to load Pb^2+^ (with K_2_PdCl_4_ type) and pH-sensitive dyes, enabling pre-concentration of C_2_H_4_ and thereby enhancing detection sensitivity. In the Pb^2+^-dye/NH_2_-UiO-66 composites, C_2_H_4_ reduced the Pb(ii) to Pb(0), generating HCl, which altered the colour of the dyes. Eight different dye-loaded UiO-66-NH_2_ units produced distinct olfactory fingerprints that were analysed by DenseNet-based image classification, enabling real-time, low-cost monitoring of fruit ripeness.

MOFs with customized structures provide valuable platforms for studying host–guest interactions and structure–activity relationships, thereby guiding the design of frameworks for colorimetric detection of volatile and gaseous analytes.^133^ With the assistance of AI, pattern recognition from a sensor array^135,138^ is an affordable challenge for users, facilitating the development of various MOF-based colorimetric gas sensors.

## Overview of Zr-based MOFs colorimetric sensors: analytes, functional sites, and fabrication methods

The performance and practicality of MOF-based colorimetric sensors are largely determined by three key aspects: the choice of analytes, the design of functional sites within the MOF structures, and the fabrication strategy of the sensing materials (Table 2). These aspects are interrelated and jointly influence the sensitivity, selectivity, and reversibility of the sensors.

**Table 2:** Zr-based MOFs colorimetric sensors: analytes, functional sites and fabrication methods

Materials	Analyte	Function sites	Fabrication	Dynamic range^a^	Reversibility	Ref.
PCN-222	Acid	Porphyrin ligands	Powder	pH = 0–4	Yes	62
PCN-222	Acid	Porphyrin ligands	Aqueous dispersion	—	No	68
PCN-224 (protonated)	Base	Porphyrin ligands	Membrane	—	Yes	71
MOF-525	Acid	Porphyrin ligands	Ionogel	pH = 0–3	Yes	73
MOF-808-EDTA-M	Acid (vapours)	EDTA-M (M = Cu or Fe)	Film	Six acid vapours	Yes	75
UiO-66-NH_2_-BCP	NH_3_	Dye (BCP)	Membrane	>20 ppm	Yes	76
UiO-66-NH_2_-anthocyanin	Acid and base	Dye (anthocyanin)	Label film	pH = 2–12	Yes	78
AC@UiO-66-NH_2_	Acid and base	Dye (alizarin complexone)	Aqueous dispersion	pH = 2–11	Yes	79
AC@UiO-66-NH_2_	Acid and base	Dye (alizarin complexone)	Label	pH = 2–11	Yes	80
UiO-66-NH_2_-RhB	PFOS	Dye (RhB)	Aqueous dispersion	>0.91 nM	No	81
PCN-224	Hg^2+^	Porphyrin ligands	Aqueous dispersion	>0.1 μM	No	89
PCN-222 + PO_4_^3−^	Pb^2+^, Cd^2+^	Porphyrin ligands	Aqueous dispersion	>1 μM	No	90
RuUiO-67	Hg^2+^	Ruthenium complex	Aqueous dispersion	>12 μM	No	91
DDTC/UiO-66	Cu^2+^	DDTC	On filter paper	>7.7 nM	No	92
MIL-140(Zr)-DHTZ	AsO_4_^3−^	DHTZ (as ligand)	Aqueous dispersion	>1 nM	No	93
UiO-66-NH_2_-5Cl2HA	As^3+^	5-Chloro-2-hydroxyacetophenone	Aqueous dispersion	>0.07 ppm	Yes	95
UiO-66-Azo-Im	Cu^2+^, Ni^2+^, and Co^2+^	Amino ligands	Paper based	>0.1 mM	No	96
UiO-66-NH_2_	F^−^	Dye (FTIC)	Aqueous dispersion	>0.9 ppm	No	98
HRP-on-UiO-66-NH_2_	NO_2_^−^	Aqueous dispersion	oxTMB	5–250 μM	Yes	100
HRP-on-UiO-66-NH_2_	NO_2_^−^	Portable kit (gelatine)	oxTMB	10–400 μM	Yes	101
PCN-224 (protonated)	Biogenic amines	Porphyrin ligands	Label	—	Yes	109
UiO-66/Au NPs	Pesticides (paraoxon)	Zr_6_ clusters	Aqueous dispersion	0.68 μg mL^−1^	No	112
UiO-66-NH_2_@TBTA COF	Glutathione	Amino ligands	Aqueous dispersion	>0.14 μM	No	115
Ab2@AgPt/PCN-223-Fe	Bacteria (*E. coli* O157:H7)	Antibodies	Paper based	276 CFU mL^−1^	No	118
PCN-222–mAb	Veterinary drug residues (CPAOZ)	Antibodies	Paper based	1.0 ng mL^−1^	No	119
PtNPs@UiO-66-NH_2_	Fusarium toxins	Paper based	PtNPs	0.15–5 ng mL^−1^ (ZEN) 0.6–10 ng mL^−1^ (FB)	No	120
PCN-222@liposome-LFIA	Veterinary drug residues (CPAOZ)	PCN-222	Paper based	0.05–50 μg g^−1^	No	121
Dye@UiO-66-Br/chitosan	Amine gases (biogenic amines)	Dye (Zn-TCPP, methyl red and curcumin)	Casting sensor array	>40.7 ppm	No	123
UiO-66-anthocyanins	Biogenic amines	Dye (anthocyanins)	Casting sensor array	>50 ppm	No	124
GOx-on-PCN-222(Fe)	Glucose	Enzyme (GOx)	Paper based	>0.25 mM	No	129
GOx-on-PCN-222(Fe)	Glucose	Enzyme (GOx)	Aqueous dispersion	>0.28 μM	No	130
GOx-on-HP-PCN-222(Fe)	Glucose	Enzyme (GOx)	Aqueous dispersion	>20 μM	No	131
Ce/Zr-UiO-66	Chloramphenicol	Paper based	Enzyme (HRP)	0.1–5000 ng mL^−1^	No	132
UiO-66(7-azaindole)	Gas (alkane)	Zr_6_ clusters	Powder	—	No	133
UiO-66-(NO_2_)_2_	H_2_S	Nitro ligands	Aqueous dispersion	>14.2 μM	No	135
Cu-MOF-808	H_2_S	Cu(ii)	Powder	>100 ppm	No	136
PCN-222(M)@PDMS	Gas (vapour)	Porphyrin ligands	Membrane sensor array	—	No	137
Pb^2+^-dye/NH_2_-UiO-66	Ethylene	Pb(ii) + dye	Membrane sensor array	>25 ppm	No	138

a In this table, we report the dynamic range as we intentionally prefer to focus on experimentally demonstrated sensing responses.

From the perspective of analytes, the Zr-based MOF colorimetric sensors covered in this review mainly focus on these four aspects: acid and base, aqueous ions, biomolecules, and volatile and gaseous analytes. In particular, some studies involving food safety monitoring and the detection of volatile and gaseous analytes show considerable application potential.^75,109,112,119,121,123,124,133,137^ These reports offer innovative strategies and ideas to address many challenges of practical interest, with recent studies further highlighting the functional versatility of Zr-based MOF systems in sensing applications.^139–143^ However, for some analytes, such as acids, mature materials already exist, like pH paper strips, which are usually cheap and convenient. Therefore, there is limited incentive to develop new and expensive alternatives. In contrast, for some analytes, such as volatile biogenic amines, these MOF colorimetric sensors represent a step forward, even if they are chemically based on the same principle. It should be noted that the selectivity in these Zr-based MOF colorimetric systems is largely governed by the sensing mechanisms rather than analyte categories alone. In practical applications, similar functional sites may interact with different analytes under various conditions, resulting in potential cross-sensitivity.

Beyond analyte selection, and in close connection with the sensing mechanism, the rational design of functional sites within MOFs plays a crucial role in achieving efficient and specific sensing responses. Functional sites, located either on the organic ligands or metal clusters^68,70,73,89,90,96,109,115,133,135,137^ that can interact with those analytes, have been incorporated into MOF structures, reflecting a deep understanding of the relationship between structure and properties in MOFs. Although some functional units are not suitable for direct incorporation into frameworks, they can be combined with MOF structure by post-modification or adsorption, with the porous structure within the MOFs providing both protection and support. However, a few studies involve overly complex combinations of multiple functional materials, without fully utilizing the advantages of the intrinsic porous structure of MOFs.

In addition to analyte and framework design, fabrication methods strongly affect the practical applicability of MOF-based colorimetric sensors. Many systems are used as aqueous dispersions, accounting for *ca.* 40% of the examples in this review. Although they can serve as assays, this format is neither cheap nor environmentally friendly. MOF particles in aqueous dispersion are typically discarded after use, especially when the colorimetric response is irreversible. Otherwise, they have to be regenerated, which usually requires adding reagents, separation by centrifugation, and repeated washing, which are time consuming and increase the overall cost. As mentioned before, according to the definition of a sensor,^16^ aqueous dispersions cannot strictly be regarded as sensors, but rather as proof-of-concept assays. This difference in technological readiness indicates the gap between fundamental development and practical application. Therefore, for more practical applications, efforts should focus on developing sensing materials based on immobilized MOF particles. When MOF particles are fabricated as test papers or smart labels,^70,75,76,96,109,119,129^ which resemble sensors more than a pile of powder or a vial of aqueous dispersion, they exhibit greater tunability and convenience. In addition, the development of artificial intelligence offers great potential for chemical colorimetric sensors, especially when combined with deep convolutional neural networks (DCNNs) and machine learning, providing rapid discrimination and accurate interpretation of the complex signals from colorimetric sensor arrays.^123,124,137,138^

## From probing materials to sensors

As discussed above, while some colorimetric assays based on MOF powders or dispersions can provide qualitative responses, these forms often lack practical usability and, if implemented in different devices, reproducible sensing performances. To achieve reliable colorimetric sensors, it is desirable to transform responsive probes, MOF powdery materials, or dispersions into integrated sensing platforms. In particular, depending on the colorimetric application, MOFs can be deposited as thin films or fabricated as membranes or patterned structures, each offering unique advantages for colorimetric sensing.^70^

MOF film deposition represents a common and practical way to transform colorimetric responsive probing materials into sensors.^144^ MOF films can be prepared by simple physical deposition methods such as spin coating, dip coating, or spray coating, where the MOF powders or dispersions are uniformly distributed on solid substrates.^70^ Alternatively, techniques involving the controlled growth of MOF directly on substrates include liquid-phase epitaxy (LPE),^145,146^ layer-by-layer (LbL),^147,148^ seeding,^149^ electrochemical,^150^ and gas phase^151^ growth that allows direct crystallization of MOFs on functionalized surfaces with improved thickness control and adhesion. These approaches produce rather uniform and stable optical interfaces, enabling reliable colour identification and facilitating integration with optical sensing devices.^152^

MOF membranes are an alternative and effective way to translate colorimetric responsive materials into sensors.^153^ MOF membranes can be prepared by *in situ* growth on porous or flexible supports,^154^ or *via* seed-assisted secondary growth to form continuous and well-adhered layers.^155^ In mixed-matrix membranes (MMMs),^156^ where pre-formed MOF particles or nanosheets are embedded in polymeric or composite matrices, the polymeric component offers enhanced mechanical stability and processability. Membranes constructed from ultrathin MOF layers provide short transport pathways, high permeability, and rapid analyte diffusion.^157^ Collectively, MOF-based polymeric membranes can combine optical transparency, structural stability, and efficient mass transport, making them particularly suitable for colorimetric sensing applications.

Although various functional coating materials have already shown promising properties, further effort is required to integrate them into miniaturized functional devices, and an essential step toward such integration is the patterning of these materials.^158^ For MOFs, patterning consists either of arranging MOF particles into defined patterns or of growing MOFs *in situ* on solid substrates. Patterning of MOF particles, for example *via* photolithography and imprinting,^159,160^ allows precise spatial localization on solid substrates and enables the fabrication of ordered arrays suitable for multi-analyte colorimetric sensing. *In situ* growth on patterned regions can be, in principle, achieved by established MOF patterning methods, such as spatially controlled growth of MOFs from dissolved precursors,^161,162^ conversion from positioned insoluble metal-based precursors,^163^ or from patterns of nucleating agents that trigger localized crystal growth.^164^ The systematic exploration of these approaches for Zr-based MOFs would enable the integration of the aforementioned robust MOF materials for the fabrication of colorimetric sensors.

Overall, the fabrication of films, membranes, and patterned MOF structures will convert dispersed colorimetric responsive MOF materials into stable, functional interfaces suitable for practical colorimetric sensing applications (Fig. 17).

## Conclusions and prospects

Zr-based MOFs, synthesized mainly through solvothermal or microwave-assisted methods, and further processed into practical platforms such as paper-based devices,^96,118–121,129,136^ labels or films,^75,78,80,109^ sensor arrays^137,138^ and hybrid membranes,^70,76^ have been widely employed as colorimetric sensors for detecting acid/base species, aqueous ions, biomolecules, and gases in both liquid and gas phases. Building on these advances, different scientific disciplines are increasingly converging to develop breakthroughs that enable rapid, real-time, user-friendly, sensitive, and cost-effective continuous monitoring across critical areas such as environmental monitoring, food and drinking water safety, and public health. The commercial implementation of colorimetric sensors based on Zr-MOFs remains limited by challenges such as long-term stability, material availability, and high development and manufacturing costs. Moreover, some applications of Zr-based MOFs as colorimetric sensors rely entirely on functional dye molecules, without making full use of the fundamental advantages of the MOF framework due to limited structural understanding. In contrast, the most advanced colorimetric applications are built upon careful MOF structure engineering and device design.

Future development of Zr-based MOF colorimetric sensors may focus on porphyrin MOFs such as PCN-224, PCN-222, and MOF-525, where porphyrin ligands enable specific colorimetric responses toward ions, gases, and vapours. Beyond porphyrins, the use of other ligands with extended π-conjugation systems to build novel MOFs and systematically investigate their optical properties also presents an exciting opportunity.

With the rapid development of characterization techniques and computational modelling, our understanding of host–guest interactions in MOF materials continues to deepen,^165^ providing a basis for the rational design of more complex and efficient colorimetric sensors. Furthermore, with advances in scalable synthesis, innovative device architectures, and big data analysis, especially when combined with AI, these sensors are expected to overcome existing limitations and offer significant potential for practical applications in environmental monitoring, food safety, and public health protection.

## Conflicts of interest

There are no conflicts to declare.

## Data Availability

No primary research results, software or code have been included and no new data were generated or analysed as part of this review.
